# Taurine deficiency damages retinal neurones: cone photoreceptors and retinal ganglion cells

**DOI:** 10.1007/s00726-012-1273-3

**Published:** 2012-04-04

**Authors:** David Gaucher, Emilie Arnault, Zoé Husson, Nicolas Froger, Elisabeth Dubus, Pauline Gondouin, Diane Dherbécourt, Julie Degardin, Manuel Simonutti, Stéphane Fouquet, M. A. Benahmed, K. Elbayed, Izzie-Jacques Namer, Pascale Massin, José-Alain Sahel, Serge Picaud

**Affiliations:** 1 INSERM, U-968, Insitut de la Vision Retinal Information Processing: Pharmacology and Pathologies, 17, rue Moreau, 75012 Paris, France; 2 UPMC Univ Paris 06, UMR_S968, Institut de la Vision, 75012 Paris, France; 3 CNRS, UMR 7210, Institut de la Vision, 75012 Paris, France; 4 Service d’ophtalmologie du Nouvel Hôpital Civil, Strasbourg, France; 5 Université de Strasbourg, UMR7237, Strasbourg, France; 6 Université de Strasbourg, UMR7177, Strasbourg, France; 7 Department of Biophysics and Nuclear Medicine, Hôpitaux Universitaires de Strasbourg, Strasbourg, France; 8 Hôpital Lariboisière, Paris, France; 9 Centre Hospitalier National d’Ophtalmologie des Quinze-Vingts, Paris, France; 10 Institute of Ophthalmology, University College, London, UK; 11 Fondation Ophtalmologique Adolphe de Rothschild, Paris, France

**Keywords:** Retina, Taurine deficiency, Ganglion cells, Autofluorescence, Degeneration, Retinal pigment epithelium

## Abstract

In 1970s, taurine deficiency was reported to induce photoreceptor degeneration in cats and rats. Recently, we found that taurine deficiency contributes to the retinal toxicity of vigabatrin, an antiepileptic drug. However, in this toxicity, retinal ganglion cells were degenerating in parallel to cone photoreceptors. The aim of this study was to re-assess a classic mouse model of taurine deficiency following a treatment with guanidoethane sulfonate (GES), a taurine transporter inhibitor to determine whether retinal ganglion cells are also affected. GES treatment induced a significant reduction in the taurine plasma levels and a lower weight increase. At the functional level, photopic electroretinograms were reduced indicating a dysfunction in the cone pathway. A change in the autofluorescence appearance of the eye fundus was explained on histological sections by an increased autofluorescence of the retinal pigment epithelium. Although the general morphology of the retina was not affected, cell damages were indicated by the general increase in glial fibrillary acidic protein expression. When cell quantification was achieved on retinal sections, the number of outer/inner segments of cone photoreceptors was reduced (20 %) as the number of retinal ganglion cells (19 %). An abnormal synaptic plasticity of rod bipolar cell dendrites was also observed in GES-treated mice. These results indicate that taurine deficiency can not only lead to photoreceptor degeneration but also to retinal ganglion cell loss. Cone photoreceptors and retinal ganglion cells appear as the most sensitive cells to taurine deficiency. These results may explain the recent therapeutic interest of taurine in retinal degenerative pathologies.

## Introduction

Taurine is a free amino sulfonic acid present in high quantities in the central nervous system (Huxtable 1989). It does not contain a carboxyl group as other amino acids entering into the composition of proteins. In the retina, it represents nearly half of the free amino acid contents (Macaione et al. 1974) reaching concentrations as high as 50 μmol/g (Voaden et al. 1977). The source of taurine is mostly exogenous, its uptake relying on very efficient transport systems exhibiting a high retinal uptake index (26.6 % in serum) (Tornquist and Alm 1986). These transport systems are expressed at the level of the hemato-retinal barrier in the retinal pigment epithelium (RPE) (Lake et al. 1977; Voaden et al. 1977; Hillenkamp et al. 2004) and in the vascular endothelial cells (Tomi et al. 2008). In retinal cells, taurine uptake was demonstrated in photoreceptors, retinal ganglion cells, retinal glial cells and in the retinal pigment epithelium cells (Lake et al. 1977; Voaden et al. 1977; Pow et al. 2002; Hillenkamp et al. 2004). A taurine high-affinity Na/Cl transporter (Slc6a6 or TauT) is expressed in these different cell types (Vinnakota et al. 1997; Pow et al. 2002; Tomi et al. 2008). However, this transporter can also take up different natural molecules including GABA and β-alanine (Tachikawa et al. 2009).

Though the exact role of taurine in the retina is not fully understood, numerous studies have demonstrated the importance of its functional and anatomical implication in the neuroretinal homeostasis (Militante and Lombardini 2002). Indeed, taurine participates in the cyclic GMP-gated channels activation, which is a key step in the phototransduction signalling cascade (Militante and Lombardini 1998). Many studies have also reported that taurine had a protective effect on cells from neuroretina (Louzada et al. 2004) and RPE (Udawatte et al. 2008). The exact mechanism of this protective effect is not well known. Activation of GABA_A_ receptors through taurine binding may decrease neuronal vulnerability to excitotoxic damage (Louzada et al. 2004). Moreover, taurine supplementation in rats has demonstrated to reduce neuronal and glial cell death in different pathological conditions (Zeng et al. 2009; Jammoul et al. 2009, 2010). In the retina, decreased taurine uptake was also found to induce retinal degeneration indicated by disorganization of the outer nuclear layer (ONL) with photoreceptor damage and eventually cell death (Hayes et al. 1975; Schmidt et al. 1976; Anderson et al. 1979; Barnett and Burger 1980; Lake and Malik 1987; Imaki et al. 1993, 1998; Leon et al. 1995). In some of these studies, some retinal dysfunction was also evidenced by eletroretinogram measurements (Jacobson et al. 1987; Quesada et al. 1988; Shimada et al. 1992).

Recently, we have attributed the retinal toxicity of the antiepileptic drug, vigabatrin, a blocker of the GABA-transaminase, to a taurine depletion (Jammoul et al. 2009). Among photoreceptors, we observed that cones were preferentially damaged (Duboc et al. 2004). More surprisingly we found that, as in patients, retinal ganglion cells are also affected by the treatment and the cell loss is correlated to the taurine depletion (Jammoul et al. 2010).

To investigate whether taurine depletion is truly responsible for damages to both cone photoreceptors and retinal ganglion cells, we have re-examined the retinal lesions produced by guanidoethane sulfonate (GES), a well-known taurine uptake inhibitor.

## Experimental methods

### Animal treatment

Mice, 8-week-old male BALB/cJRj (Janvier, Saint Isle, France), were used and handled according to the principles of the ARVO statement for the use of animals in ophthalmic and vision research. Control mice were maintained on a standard diet and water ad libitum, under 12/12 h light/dark cycle, while treated mice were given GES (Toronto research chemical inc., North York, ON, Canada) in drinking water at a concentration of 1 % for 2 months. The incidence of the treatment on mice development was estimated by weighing the animal at the beginning of the study and before killing.

### Retinal electrophysiology

Electroretinograms (ERG) were performed on all animals 2 months after GES treatment introduction. Animals were dark adapted for 12 h prior to the recording. They were anaesthetized with an intra-peritoneal injection (10 μl/g) of a mixture of ketamine (10 %, Virbac, France) and xylazine (7.5 %, Bayer, Germany) diluted in a 0.9 % NaCl solution. Corneas were anaesthetized with oxybuprocaïne chlorhydrate (0.4 %) (Thea Lab., France) and the pupil dilated with tropicamide (0.5 %, Thea Lab). Each animal was placed on a heating pad; the eyelids were retracted so the eye could be maintained open during the recording. A gold electrode was placed on the cornea with a drop of methylcellulose (Ocry-gel, Thérapeutique Vétérinaire Moderne, France) while the neutral and the reference electrodes were placed on the tail and on the head of the animals, respectively. Light stimulations were delivered in a Ganzfeld with flash intensities ranging from 10^−4^ to 10 cd s m^−2^. Animals were then light adapted for 10 min with a background light of 25 cd m^−2^. The background ganzfeld stimulus was produced by rear illumination of a white diffuser (35 mm in diameter). Oscillatory potentials (OPs) were isolated for a light stimulus of 3 cd s m^−2^ by filtering the responses with high- and low-pass filters at 100 and 300 Hz, respectively. Amplitudes and latencies of the scotopic ERG a- and b-waves were measured at the maximum negative and positive peaks of the recordings with respect to the baseline before the stimulation. OPs amplitudes were measured from the negative peak to the next positive peak. For each recording, the sum of the amplitudes of the first three OPs was calculated and used for statistical analysis. For photopic ERGs recordings, animals were subjected to light flashes and background light; the light intensity of the flash was 10 cd s m^−2^. Ten recordings with an interstimulus interval of 30 s were averaged. Flicker light stimulation at 15 Hz was performed and responses were averaged during 40 s of stimulation. Responses were amplified, high- and low-pass filtered (1–300 Hz) and digitised (Multiliner Vision, Toennies/Jaeger, Hoechberg, Germany). Amplitudes and latencies of the photopic ERG b-wave were measured at the maximum negative and positive peaks of the recordings with respect to the baseline before the stimulation.

### Ocular examination, funduscopy and fluorescein angiographs

Fundus examinations and angiographs were performed on all mice 2 months after initiating the GES treatment. Cornea, eye lens and fundus were examined with a biomicroscope (BQ, Haag-Streit, Germany) and a 90-dioptre lens (Volk, USA). Angiographs were performed using a scanning laser ophthalmoscope (SLO) with an excitation light at 488 nm (HRA, Heidelberg, Germany). Retinal angiography was performed following a 25 % fluorescein intraperitoneal injection in a 0.9 % NaCl solution. Mouse pupils were dilated with an ocular instillation of tropicamide eye drop (0.5 %). Images of the fundus were collected on each eye before, 1 and 5 min after the fluorescein injection. For each animal, best autofluorescence images were obtained by computing with the Heidelberg HRA software a mean image out of four.

### Cell labelling

Eye cups were fixed overnight at 4 °C in 4 % (wt/vol) paraformaldehyde in phosphate-buffered saline (PBS; 0.01 M, pH 7.4). The tissue was cryoprotected in successive solutions of PBS containing 10, 20 and 30 % sucrose at 4 °C, oriented along the dorso-ventral axis and embedded in NEG50^®^ (Microm, Francheville, France). Retinal sections (8–10 μm thickness) were treated for 5 min in PBS containing 0.1 % Triton X-100 (Sigma, St. Louis, MO, USA), rinsed and incubated in PBS containing 1 % bovine serum albumin (Sigma), 0.05 % Tween 20 (Sigma) for 1 h at room temperature. The primary antibody added to the solution was incubated overnight at 4 °C. Polyclonal antibodies were directed against rabbit glial fibrillary acidic protein (GFAP) (1:100, Dako, Glostrup, Denmark), rabbit PKC alpha (c-20 sc 208, 1:1,000, Santa Cruz, CA, USA) and rabbit GABA (YY100, 1:100, Signature Immunologics, Salt Lake City, UT, USA). Monoclonal antibodies were directed against mouse Goα (1:200, Chemicon, Billerica, MA, USA), mouse Calretinin (6B8.2, 1:1,000, Millipore, Billerica, MA, USA) and mouse Brn-3a (1:100, Chemicon). Sections were rinsed and then incubated with the secondary antibody, goat anti-rabbit IgG or goat anti-mouse IgG conjugated to either Alexa TM594 or Alexa TM488 (1:500, Molecular Probes, Carlsbad, CA, USA) for 1 h. Inner/outer segments of cone photoreceptors were stained with a peanut lectin (PNA, 1:40, Molecular Probes) overnight at 4 °C. Cell nuclei were revealed incubating the specimens with 4′, 6-diamidino-2-phenylindole (10 μg/mL, DAPI, Sigma-Aldrich) in the last incubated solutions for 1 h at room temperature. Sections were rinsed and mounted with Permafluor^®^ reagent (Microm).

### Cell quantification

PNA, Brn-3a and GFAP labelled sections were viewed with a fluorescent microscope (Leica DM5000B, Germany) mounted with a digital camera (Coolsnap fx, Photometrix, Roper Scientific, USA) linked to a computer running image analysis software (Metavue 6.2R4, Universal Imaging Corp, USA). Digital images were acquired using identical settings. GFAP immunostaining was used for detection of retinal areas with reactive gliosis. Quantifications of the cone photoreceptors and ganglion cells were performed on two different retinal sections for each animal including the optic nerve and oriented along the dorso-ventral axis. Cone photoreceptors were counted following PNA labelling to visualise their inner/outer segments while Brn-3a labelling was used for the detection of ganglion cells. Cellular densities were calculated using the ratio between the mean cell number and the mean length of the both entire sections (Wright Cell Imaging Facility, ImageJ software, National Institutes of Health, Bethesda, MD, USA).

Amacrine and GABA labelled sections were acquired with the Nanozoomer 2.0HT (Hamamatsu Photonics, Japan) using identical settings and a 20× objective for all sections with the 556/613 nm excitation/emission filters. Images of DAPI-stained nuclei were obtained using the 387/433 nm excitation/emission filters. NDPView Nanozoomer associated software was used for quantification of amacrine and GABA-positive cells. Cellular densities were calculated using the ratio between the mean cell number and the mean length of the both entire sections.

### Autofluorescence analysis

Autofluorescence digital images were acquired with the Nanozoomer 2.0HT (Hamamatsu Photonics, Japan and NDPView Nanozoomer associated software) using identical settings and a 20× objective for all sections with the 556/613 nm excitation/emission filters. Images of DAPI-stained nuclei were obtained using the 387/433 nm excitation/emission filters.

For each section, four distinct 50 × 50 μm representative areas of the dorso-ventral axis of the retina were analysed at the RPE location: dorso-peripheral (DP), dorso-central (DC), ventro-central (VC) and ventro-peripheral (VP).

Mean autofluorescence along the RPE axis on a 50 × 50 μm area was measured by Matlab software (v 7.7.0 R2008b, MathWorks™, MA, USA) and curves were centred at the RPE level. Autofluorescence of photoreceptors outer segments next to RPE cells was used as reference value corresponding to arbitrary unit zero. Statistical analysis was performed on max RPE autofluorescence values.

### Taurine blood concentration measurements

Blood samples were collected in haemolysis tubes containing heparin (14 IU mL^−1^) and centrifuged (2,200 g, 15 min). Plasma amino-acid analysis was performed by ion exchange chromatography with nihydrin detection using a JEOL AMINOTAC analyser (Pasteur-Cerba, Paris, France).

### Taurine retinal concentration using HRMAS-NMR (High-resolution magic angle spinning nuclear magnetic resonance) measurements

In order to ascertain that retinal taurine content was reduced after GES treatment, retinas from treated (*n* = 8) and untreated animals (*n* = 8) were collected after 1 month of GES treatment and stored at −20 °C.

Each retina was prepared as previously described (Piotto et al. 2008) by introducing the frozen tissue into a disposable 30 μl KelF insert at −20 °C.

HRMAS spectra were recorded on a Bruker Avance III 500 spectrometer (Bucker BioSpin, Billerica, MA, USA) operating at a proton frequency of 500.13 MHz. The spectrometer was equipped with a 4-mm double resonance (^1^H, ^13^C) gradient HRMAS probe. A Bruker Cooling Unit was used to regulate the temperature at 4 °C by cooling down the bearing air flowing into the probe. All NMR experiments were conducted on samples spinning at 3,502 Hz to keep the rotation sidebands out of the spectral region of interest.

For each biopsy sample, a one-dimensional proton spectrum using a Carr-Purcell-Meiboom-Gill (CPMG) pulse sequence was acquired as previously reported (Piotto et al. 2008).

Metabolite assignments were identified using standards metabolites chemical shift tables available in the literature (for taurine 3.4416 and 3.388 ppm). Spectra were normalized according to sample weight. Quantification of taurine was performed automatically by an in house programme using MATLAB 7.0 (Mathworks, Natik, USA). Taurine peak integration was then compared with the one obtained on the lactate reference and were corrected according to the number of protons. Results were expressed in nmol/mg of tissue.

### Statistical analysis

Statistical comparisons were made by unpaired *t* test when possible and otherwise by non parametric Mann–Whitney *U* test. Computer software was used for all analysis (Statview 5.0, SAS Institute Inc., USA). Significant tests were performed with α = 0.05.

## Results

**Fig. 1:**
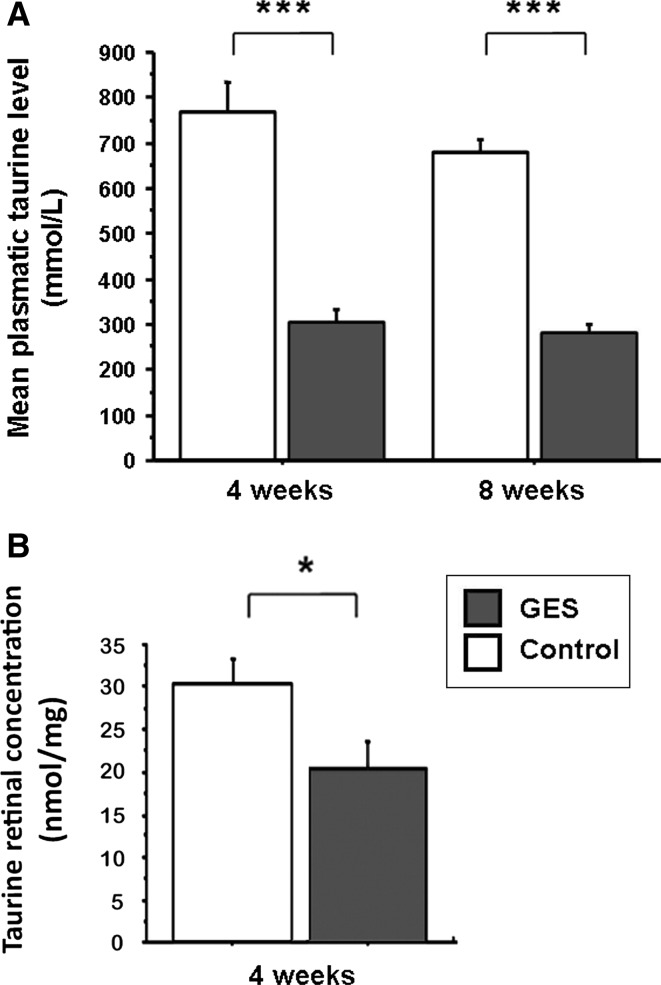
Taurine deficiency in GES-treated mice. Plasma taurine levels were measured in control animals and in GES mice after 1 and 2 months of treatment (*n* = 8, SEM, *P* < 0.001, *triple* *asterisks* denote Mann–Whitney test) (**a**). Taurine concentration in retinal tissue was also reduced after 1 month of treatment (*n* = 8, SEM, *P* = 0.046, *asterisk* denotes Mann–Whitney test) (**b**)

**Fig. 2:**
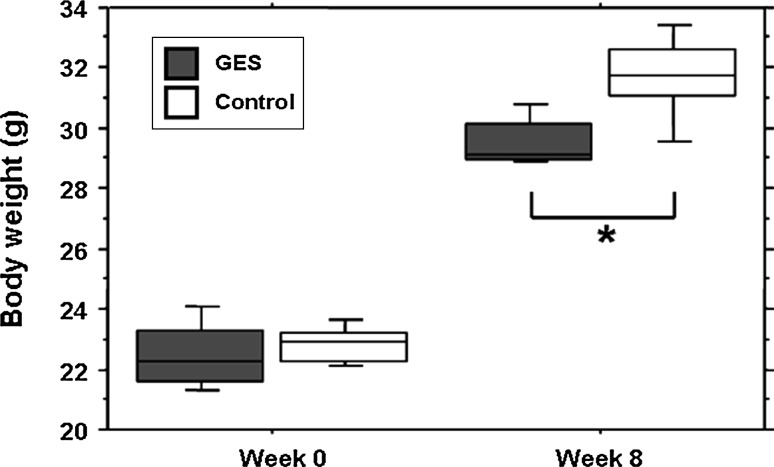
The weight development is reduced in GES-treated mice. No difference in the mean weight is noted at the beginning of the study (week 0), between the treated group and the control group (SEM, *n* = 8, *P* > 0.05, Student’s *t* test). After 2 months of treatment, the mean weight of the GES-treated mice is significantly reduced (SEM, *n* = 8, *P* = 0,024, *asterisk* denotes Student’s *t* test)

When mice were administered GES (1 %) in their drinking water, their taurine plasma level was significantly decreased at 1 month (GES group: 305.75 ± 27.13 μmol L^−1^, SEM, *n* = 8; control group: 770.62 ± 62.47 μmol L^−1^, SEM, *n* = 8, *P* < 0.001) and 2 months (GES group: 283.12 ± 15.35 μmol L^−1^, SEM, *n* = 8; control group: 681.5 ± 27.51 μmol L^−1^, SEM, *n* = 8, *P* < 0.001) (Fig. 1a). At that point, the weight development was also significantly reduced in GES-treated mice (GES group: 29.54 ± 0.28 g, SEM, *n* = 8; control group: 31.67 ± 0.51 g, SEM, *n* = 8, *P* = 0.024), while no difference was noted at the beginning of the study (GES group: 22.46 ± 0.38 g, SEM, *n* = 8; control group: 22.84 ± 0.21 g, SEM, *n* = 8, *P* > 0.05) (Fig. 2). Taurine retinal concentration was also reduced in GES mice from 1 month after GES treatment (GES group: 20.33 ± 3.42 nmol/mg, SEM, *n* = 8; control group: 30.51 ± 2.93 nmol mg, SEM, *n* = 8, *P* = 0.046) (Fig. 1b)

**Fig. 3:**
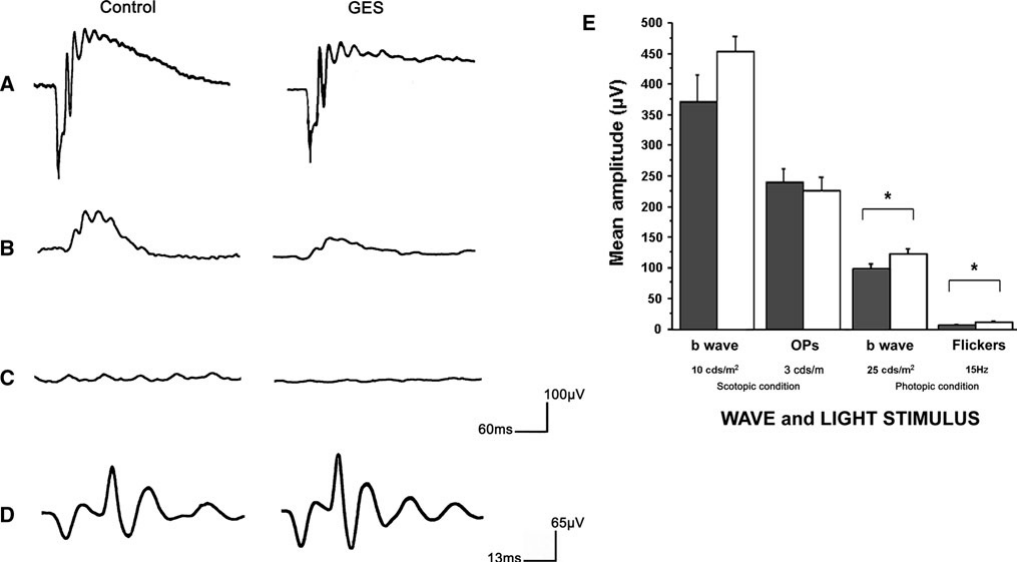
Retinal cell function in GES-treated mice. Electroretinogram (ERG) response of a 10 cds m^−2^ scotopic flash light stimulus recorded in a control animal, and in GES mouse (**a**). Response of a 25 cds m^−2^ photopic flash light stimulus recorded in a control animal, and in GES mouse (**b**). Photopic ERG response to a 15-Hz flickers light stimulus in a control animal, and in GES mouse (**c**). Oscillary Potentials (Ops) isolated in a control, and a GES mouse (**d**). Quantification of scotopic and photopic ERG amplitudes in controls (*n* = 15), and GES-treated animals showing the significant decrease in standard flash and flickers photopic ERG amplitudes. (SEM, *n* = 8, *P* < 0.05, *asterisk* denotes Student’s *t* test) (**e**)

To determine whether the GES treatment and its consequent taurine plasma and retina level decrease induced retinal dysfunction, scotopic and photopic electroretinograms (ERGs) were performed on both treated and untreated animals. Scotopic ERG recordings were made in dark adapted animals (24 h of dark adaptation) with flashes of different light intensities (0.1, 1, 100, 1,000 and 10,000 mcds m^−2^). In these recordings, the first negative signal (a-wave) provides an in vivo measurement of rod photoreceptor light response dynamic whereas the consecutive positive signal (b-wave) is informative about the postsynaptic bipolar cells. Although cones can be activated at certain light intensities, these measurements are highly rod dominated. Mean a- (data not shown) and b-wave amplitudes of scotopic ERGs were always reduced in GES animals as compared with controls. However, the differences between the two groups were not statistically significant (*t* test, *P* > 0.05) (Fig. 3a, e). When oscillatory potentials informative of the third-order neurones, namely amacrine cells, were isolated with a more restrictive filtering of the ERG recordings, no significant difference could be detected (Fig. 3d, e). In contrast, when a background light was used to saturate rod photoreceptors, the response to an intense flash, the photopic ERG, showed a significant decrease in the GES group as compared with control mice (GES group: 97.61 ± 8.74 μV, SEM, *n* = 8; control group: 122.91 ± 7.44 μV, SEM, *n* = 15, *P* = 0.048). This difference indicated that the cone pathways (cones and their postsynaptic cells) have a functional deficit. Similarly, when a flickering light was applied at a frequency incompatible with the slow dynamic of the rod photoreceptor light response, we found that amplitude of the 15 Hz flicker response was also reduced confirming a dysfunction in the cone pathway (GES group: 6.46 ± 1.89 μV, SEM, *n* = 8; control group: 11.61 ± 1.44 μV, SEM, *n* = 15, *P* = 0.045) (Fig. 3b, c, e). These measurements indicated that GES treatment and its consecutive taurine depletion induce retinal dysfunction of the cone pathway in adult mice.

**Fig. 4:**
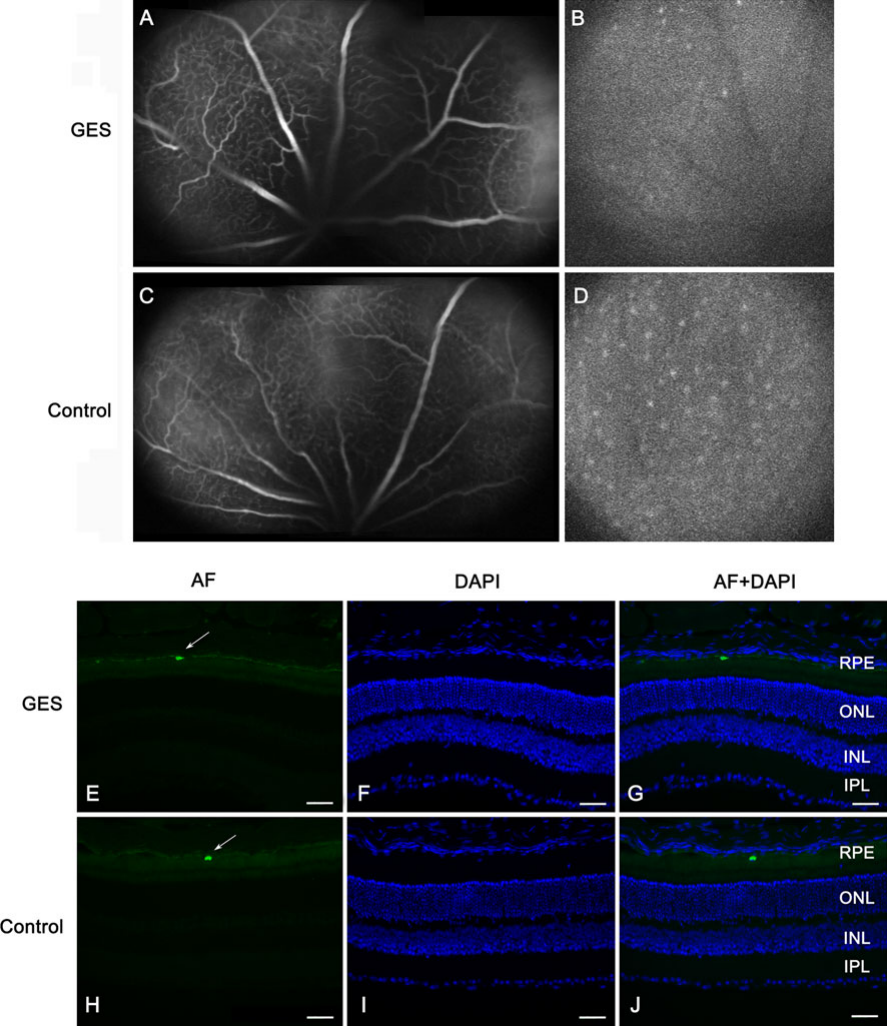
Taurine deficiency modifies retinal autofluorescence (AF) but does not alter vascular permeability in GES-treated adult mice. Vascular network seen in late phase of the angiography (**a**, **c**). No difference is detected between GES-treated mice (**a**) and controls (**c**). AF images of the fundus (**b**, **d**) revealed the presence of sparse numerous round autofluorescent dots in both controls and GES mice. The intensity and the number of these dots were lower in GES-treated mice (**b**) than in controls (**d**). AF level was analysed on eye sections stained with DAPI in GES-treated mice (**e**–**g**) and in controls (**h**–**j**): scattered autofluorescent bodies (*arrows*) were present at the level of the retinal pigment epithelium (RPE) in both groups but a general increase in the RPE fluorescence was noted in GES-treated mice, such that the fluorescent bodies offered less contrast than in controls

**Fig. 5:**
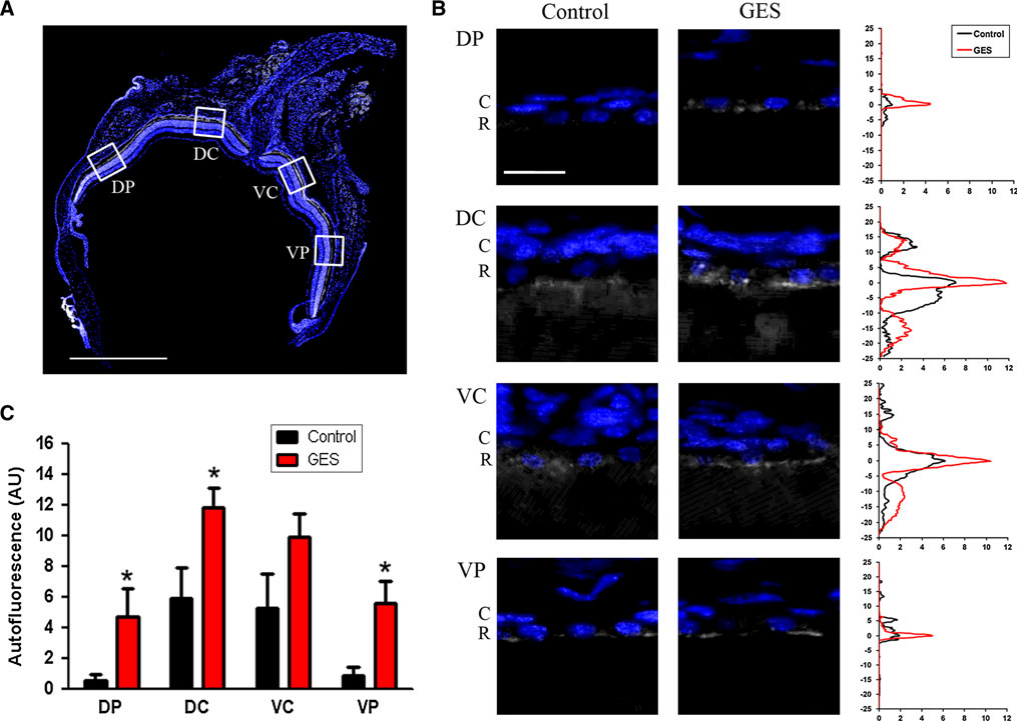
Autofluorescence in the retinal pigment epithelium (RPE). Autofluorescence at the RPE level was measured in four distinct areas on retinal sections along the dorso-ventral axis: dorso-peripheral (DP), dorso-central (DC), ventro-central (VC) and ventro-peripheral (VP). Dapi-labelled nuclei are shown in *blue* while *grey levels* correspond to the autoflorescence measured under a 556-nm excitation light. *Scale bar* represents 1 mm (**a**). Representative pictures of autofluorescence observed at the RPE level in one control and one GES-treated mouse in each area. Dapi-labelled nuclei correspond to choroid cells (*C*) and RPE cells (*R*). The corresponding autofluorescence measurement is represented on the graph at the right. Note the increase in autofluorescence observed in GES-treated animals at the RPE level. *Horizontal axis* corresponds to arbitrary units of autofluorescence and vertical axis corresponds to distance from RPE (μm). *Scale bar* represents 20 μm (**b**). Mean maximal values of autofluorescence at the RPE level according to retina area and treatment. Significant increase of autofluorescence in DP, DC and VP areas were found in GES mice (SEM, *n* = 8, *P* < 0.05, *asterisk* denotes Mann–Whitney test) (**c**)

Examination of eye fundi and angiographs were performed to look for macroscopic retinal changes, because this technique is classically used in clinical investigations to localize retinal lesions (Schmitz-Valckenberg et al. 2008). Fundi were normal in all animals. No cataract was present in the treated animals, which could have impaired the ERG recordings. No vascular damage was found on fluorescein angiographs in any animal. Interestingly, numerous peripheral autofluorescent round spots were observed in both groups. Surprisingly, their presence and number were decreased in GES-treated animals (Fig. 4a–d). To examine whether autofluorescence anomalies and retinal dysfunction were related to cellular damage, a histological examination on retinal sagittal sections was performed. Large fluorescent bodies were scattered throughout the retinal pigment epithelium in both GES-treated and control animals. The difference between the two groups appeared as an increase in the autofluorescence of the retinal pigment epithelium such that the fluorescent bodies offered less contrast in GES-treated mice (Fig. 4e–j). Furthermore, quantitative analysis of the retinal pigment epithelium in four distinct regions of the retina revealed that autofluorescence is not homogeneous along the retina (Fig. 5). In retinas of control and GES-treated mice, intensity was higher in the central region than in the periphery. The GES treatment greatly increased this autofluorescence at the periphery (seven to ninefold) and less in central areas (1.9- to 2-fold) (Fig. 5). This increase in autofluorescence was statistically significant in the dorso-peripheral area (GES group: 4.7 ± 1.9 AU, SEM, *n* = 8; control group: 0.5 ± 0.4 AU, SEM, *n* = 8, *P* = 0.029), the dorso-central area (GES group: 11.8 ± 1.3 AU, SEM, *n* = 8; control group: 5.9 ± 2.1 AU, SEM, *n* = 8, *P* = 0.04) and the ventro-peripheral area (GES group: 5.6 ± 1.5 AU, SEM, *n* = 8; control group: 0.8 ± 0.6 AU, SEM, *n* = 8, *P* = 0.029), but not in the ventro-central area (GES group: 9.9 ± 1.6 AU, SEM, *n* = 8; control group: 5.2 ± 2.4 AU, SEM, *n* = 8, *P* = 0.186).

**Fig. 6:**
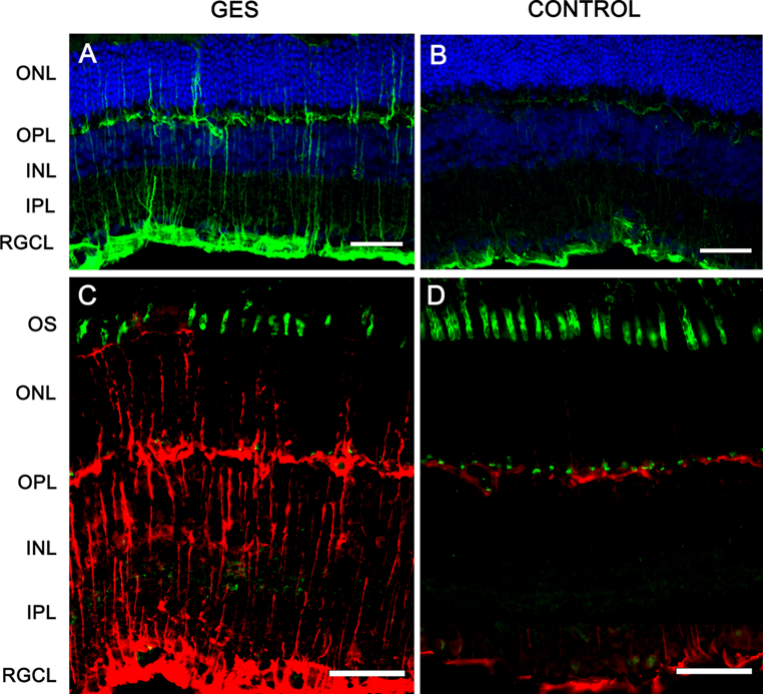
Taurine deficiency induces abnormal glial reactivity. The eyes sections were stained with DAPI and immunolabelled with anti-GFAP antibodies (**a**, **b**): glial reactivity was found in GES-treated mice as GFAP-positive processes extended vertically throughout the retina (**a**, **c**). The GFAP labelling was normally limited to the retinal ganglion cell layer and OPL in the control mice (**b**, **d**). Muller cells processes (*red*) extended to the outer limiting membrane, in contact with disorganized photoreceptors segments [stained in *green* with peanut lectin (PNA)] in GES-treated mice (**c**) as compared with controls (**d**)

The occurrence of retinal lesions is usually signed by retinal gliosis with increased expression of the GFAP (Wang et al. 2008; Jammoul et al. 2009). To assess whether retinal lesions were triggered by the GES treatment, retinal sections were therefore immunolabelled against GFAP. In control animals, GFAP expression was limited to the inner limiting membrane, whereas it extended throughout the retina in GES-treated animals from the inner limiting membrane to the outer limiting membrane (Fig. 6). These observations confirmed the presence of important retinal lesions in GES-treated animals.

**Fig. 7:**
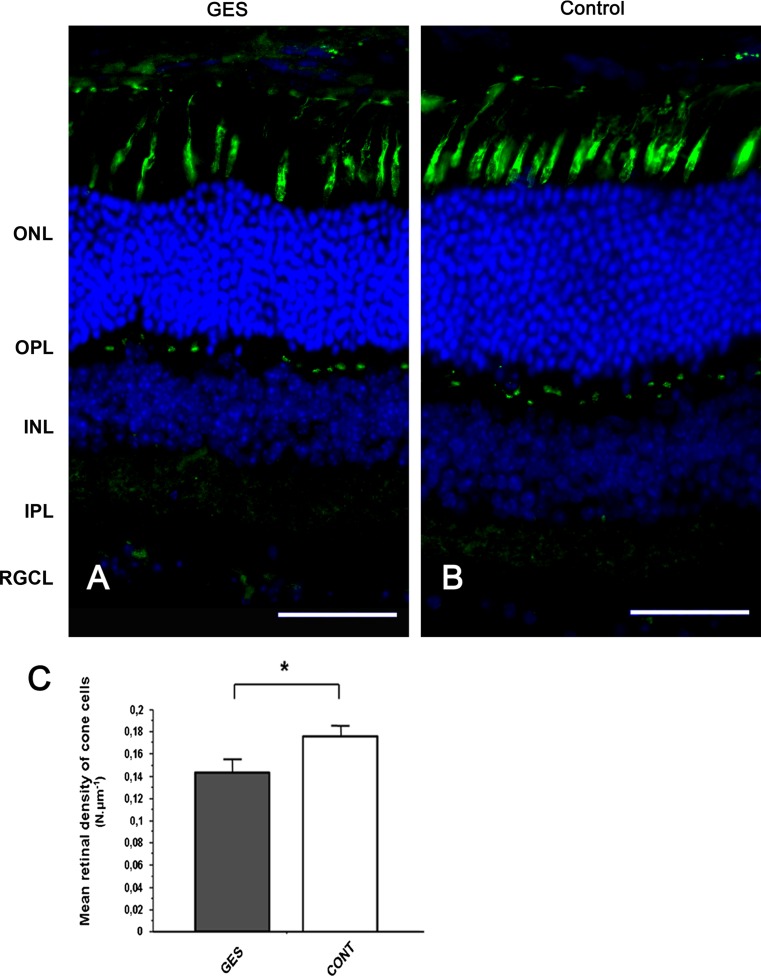
Taurine deficiency induces cone photoreceptors loss in GES-treated mice. Retinal sections of control animal (**b**) and GES-treated mice (**a**) were stained with peanut lectin (**a**, **b**), Inner and outer segments of cone photoreceptors are absent in many points of the retina in GES mice, leaving optically empty spaces throughout the segments photoreceptors line (**a**). These spaces are not detected on control eyes sections (**b**). Even if present, the inner/outer segments of cone photoreceptors seem broken and not well lined up in the treated mice (**a**) whereas they are aligned in controls (**b**). Cone photoreceptors count revealed a decrease number of cells in the GES-treated mice (SEM, *n* = 8, *P* < 0.05, *asterisk* denotes Student’s *t* test). *Scale bars* represent 25 μm (*ONL* outer nuclear layer, *OPL* outer plexiform layer, *INL* inner nuclear layer) (**c**)

**Fig. 8:**
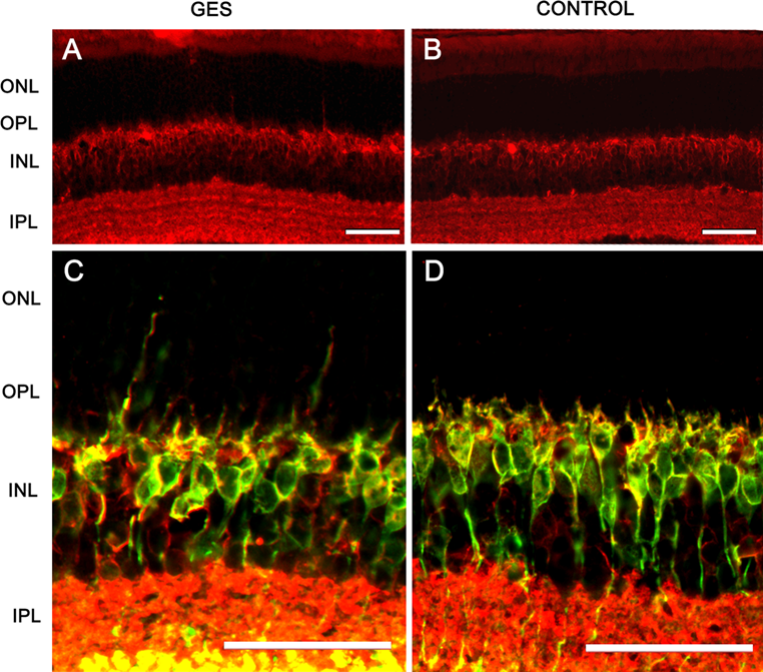
Taurine deficiency induces abnormal bipolar cell plasticity. Discrete neuronal plasticity was indicated by a few extensions of Goα immunopositive bipolar cell dendrites into the ONL in the treated group (**a**), those extensions were absent in control animals (**b**). ON rod bipolar cells were stained in green with PKCα antibody and both rod and cone ON bipolar cells were stained in red with Goα antibody (**c**, **d**): co-immunolabelled dendrites extending into the ONL were observed in GES-treated mice (**c**) and not in controls (**d**) indicating a synaptic plasticity in rod bipolar cells. *Scale bars* represent 25 μm (*ONL* outer nuclear layer, *OPL* outer plexiform layer, *INL* inner nuclear layer, *IPL* inner plexiform layer, *RGCL* retinal ganglion cell layer)

To further assess the nature of these retinal lesions, different cell types were stained or immunolabelled. When cone outer/inner segments were labelled with the peanut agglutinin lectin, they appeared more scattered in GES-treated animals (Fig. 7a, b). Their quantification along whole retinal sections indicated that the GES treatment induced a 20.4 % loss of cone outer/inner segments (Fig. 7c) (GES group: 0.140 ± 0.01 segments/μm, SEM, *n* = 8; control group: 0.176 ± 0.01 segments/μm, SEM, *n* = 8, *P* = 0.023). In some instances, a disorganization of the outer nuclear layer was also visible. When ON bipolar cell postsynaptic to photoreceptors were immunolabelled by a PKCα antibody (staining ON rod bipolar cells) and a Goα antibody (staining both rod and cone ON bipolar cells), numerous dendrites extending into the outer nuclear layer were observed and they were always co-immunolabelled identifying them as rod bipolar cells (Fig. 8). These observations indicated that rod bipolar cells undergo synaptic plasticity and formation of ectopic synapses in the outer nuclear layer.

**Fig. 9:**
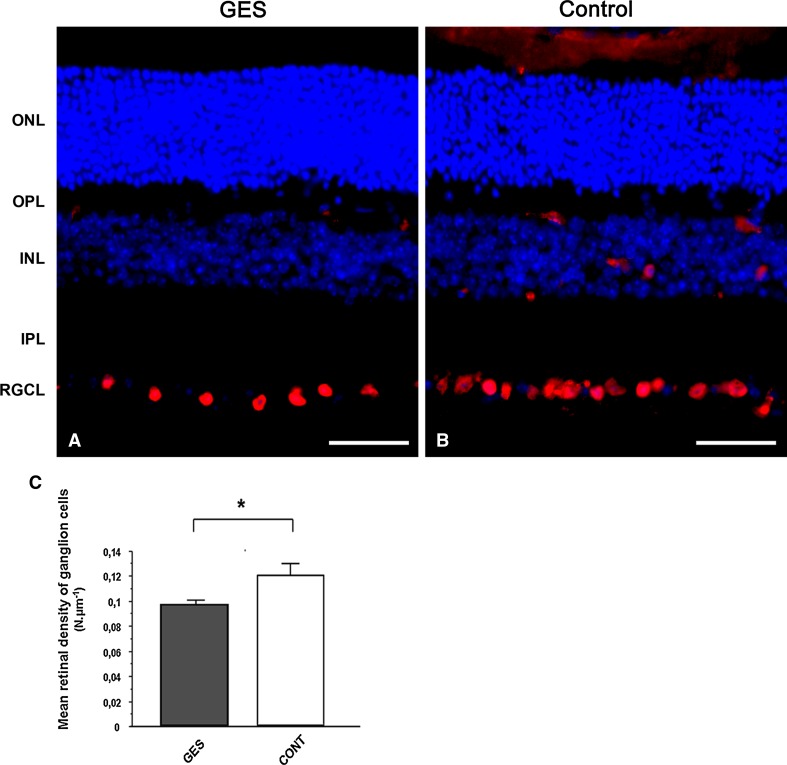
Taurine deficiency induces retinal ganglion cells loss in GES-treated adult mice. Retinal sections of GES-treated mice (**a**) and control mice (**b**) were immunolabelled with antibodies directed to Brn-3A (**a**, **b**). Ganglion cells count showed a decrease in ganglion cell density in GES-treated mice as compared with controls (SEM, *n* = 8, *P* < 0.05, *asterisk* denotes Student’s *t* test) (**c**). *Scale bars* represent 25 μm (*ONL* outer nuclear layer, *OPL* outer plexiform layer, *INL* inner nuclear layer, *IPL* inner plexiform layer, *RGCL* retinal ganglion cell layer)

Then, retinal ganglion cells were immunolabelled with an antibody directed against the transcription factor Brn-3a, which labels more than 90 % of this cell population (Nadal-Nicolas et al. 2009). Again, GES-treated mice appeared to exhibit less retinal ganglion cells than control animals (Fig. 9a, b). The retinal ganglion cell quantification on whole retinal section indicated that the GES treatment led to a 19.3 % decrease in the density of retinal ganglion cell population (Fig. 9c) (GES group: 0.096 ± 0.003 cells/μm, SEM, *n* = 8; control group: 0.119 ± 0.009 cells/μm, SEM, *n* = 8, *P* = 0.027). As GES can be taken up by the taurine transporter (TauT) (Tachikawa et al. 2009) to eventually become toxic to neurones (Hiramatsu 2003), we verified that GES was not directly toxic to retinal ganglion cells. Thus, pure rat retinal ganglion cells were prepared as described previously (Fuchs et al. 2005) and kept in culture for 6 days in the presence of GES (1 mM). In fact, their number were increased by 19.8 ± 9.8 % (SEM, *n* = 9), but this difference was not statistically significant indicating thereby that GES is not toxic to retinal ganglion cells.

**Fig. 10:**
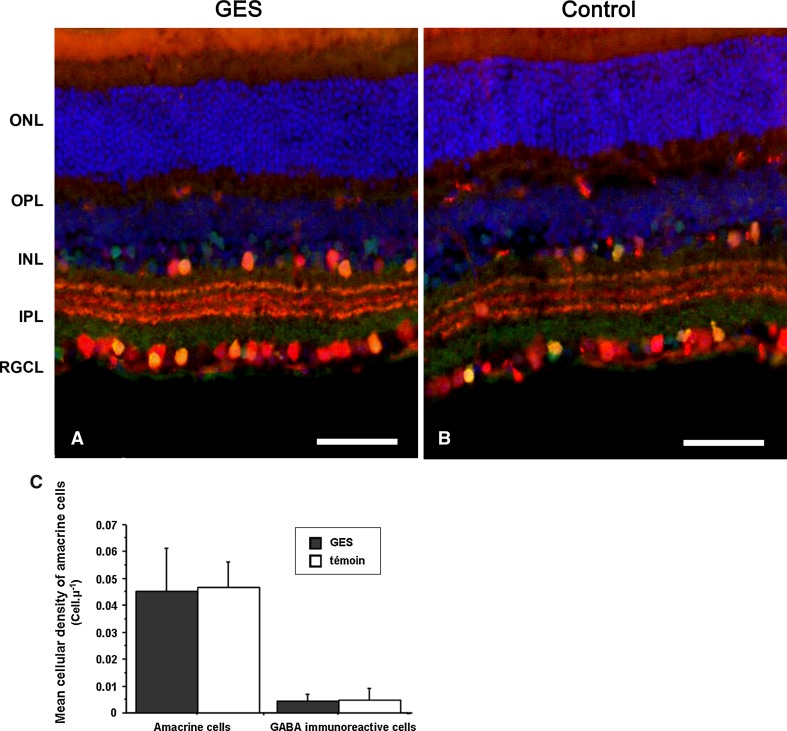
Amacrine cells were not affected in GES mice. The density of amacrine cells did not differ between treated and untreated mice. In both groups (GES, **a**) (controls, **b**), amacrine cells were stained in *red* with anti-calretinin antibodies while GABA imunoreactive cells were stained in *green* with anti-GABA antibodies. GABA imunoreactive amacrine cells were co-labelled and appeared in *yellow*. Cell count did not show any difference in the density of both types of amacrine cells between the two groups of animals (**c**). *Scale bars* represent 50 μm (*ONL* outer nuclear layer, *OPL* outer plexiform layer, *INL* inner nuclear layer, *IPL* inner plexiform layer, *RGCL* retinal ganglion cell layer)

Finally, to investigate whether the retinal ganglion cell loss is related to an unspecific degeneration of the inner retina, we quantified two populations of amacrine cells, the calretinin immunopositive cells and the GABA-containing cells. In both cases, the difference was not statistically significant either between GES mice and control animals in calretinin-positive amacrine cells (GES group: 0.045 ± 0.006 cells/μm, SEM, *n* = 8; control group: 0.047 ± 0.003 cells/μm, SEM, *n* = 8, *P* > 0.05), or in GABA immunoreactive cells (GES group: 0.004 ± 0.001 cells/μm, SEM, *n* = 8; control: 0.005 cells/μm ± 0.002 SEM, *n* = 8, *P* > 0.05) (Fig. 10). These results demonstrated that the GES treatment and likely the consecutive taurine depletion triggered retinal ganglion cell degeneration.

## Discussion

Retinal degeneration has been extensively investigated in taurine free-diet fed cats (Hayes et al. 1975; Schmidt et al. 1976; Barnett and Burger 1980; Lake and Malik 1987; Leon et al. 1995) and monkeys (Imaki et al. 1993). The taurine depletion was also induced in cats and rats by treatments with taurine transport inhibitors, such as β-alanine or GES (Pasantes-Morales et al. 1983; Imaki et al. 1998). In the GES-treated model, we observed a decrease in the taurine plasma and retinal level already detectable as soon as 1 month of treatment. This decrease is likely due to the reported GES-induced inhibition of taurine transporters (Tachikawa et al. 2009) that are expressed at the level of the intestine epithelium (Satsu et al. 1997) and in retinal capillary endothelial cells (Tomi et al. 2008). In previous reports, GES treatment consequences were characterised by photoreceptor degeneration with disruption and twisting of the discs membranes and alterations of the inner and outer segments. Photoreceptors cell loss appeared at the late stage of the disease (Hayes et al. 1975). However, in these studies generally performed during 1970–1980s, it was not possible to distinguish a relative sequence of degeneration between rods and cones. The rod photoreceptor degeneration was clear because the photoreceptor layer mostly composed of rods was severely reduced (Lake and Malik 1987). However, concerning cone photoreceptors, it was unclear whether they would undergo a secondary degeneration as a mere consequence of the rod loss as observed in retinitis pigmentosa or whether they are directly affected. At a functional standpoint, alteration of scotopic ERGs showing a decrease in a- and b-wave amplitudes has been reported in relation to rod photoreceptor damages (Cocker and Lake 1987). The fact that photopic ERGs were altered whereas statistically significant modifications of scotopic ERGs could not be found in our results, suggested that the cone pathway was more affected than the rod pathway. Quantification of cone outer/inner segments confirmed this cone damage while the maintained general structure of the retina, except occasional areas with a disorganized outer nuclear layer, was consistent with the idea that rods were less affected. However, the rod pathway was also affected as indicated by the abnormal dendritic plasticity in rod ON bipolar cells. The increase in autofluorescence of the retinal pigment epithelium may relate to the removal of the damaged structures in both rods and cones. Further studies will have to define if cone damages are occurring prior to rod degeneration.

Analysis of fundus and angiographs of the treated animals did not reveal typical retinal lesions observed in cat retinopathy following taurine deficiency (Bellhorn 1976; Barnett and Burger 1980). However, significant difference was found in autofluorescence at the RPE level between GES-treated animals and controls. Due to the lack of the antioxidant function of taurine, the autofluorescence observed at the level of photoreceptor outer segments might be a consequence of an increased oxidation when animals were exposed to ambient light. Therefore, the increase of autofluorescent materials in the RPE cells could be due to the phagocytosis of damaged photoreceptor outer segments and/or to a difficulty of the RPE to eliminate these materials that might correspond to lipofuscin. Indeed, Sparrow et al. (2010) showed that photo-oxidation of RPE lipofuscin can result in heightened fluorescence emission. Furthermore, we showed that the autofluorescence increase was more important in the dorsal area. This is consistent with previous observation where retinal lesions are usually larger in taurine-deprived animals such as those treated with vigabatrin (Duboc et al. 2004). Our findings may suggest a RPE dysfunction induced by GES treatment. This hypothesis is further supported by the abnormal oculogram in vigabatrin-treated patients (Coupland et al. 2001) who also exhibit a taurine depletion (Jammoul et al. 2009). The uptake of taurine in the RPE is well known (Peterson and Miller 1995; Hillenkamp et al. 2004) and a recent study has reported a protective effect of taurine on RPE cells (Udawatte et al. 2008). Future investigations should address the relationship between this potential RPE dysfunction and photoreceptor death under taurine depletion.

In taurine-free animals, the state of the inner retina was poorly investigated. One study mentioned a reduction in the inner plexiform layer (Imaki et al. 1998). In vigabatrin-treated animals showing a taurine depletion (Jammoul et al. 2010), we had demonstrated a major plasticity at the level of rod photoreceptor terminals (Wang et al. 2008). In our GES-treated mice, rod ON bipolar cell dendrites exhibited a similar elongation into the outer nuclear layer. Such a plasticity was also described in different animal models with mutated proteins at the rod synaptic terminals like bassoon (Specht et al. 2007), the Cacna1f gene encoding the alpha1F subunit of photoreceptor voltage-dependent calcium channels (Chang et al. 2006) and the Ca^2+^ binding protein, Cabp4 (Haeseleer et al. 2004). The formation of ectopic synapses in the outer nuclear layer as previously described in vigabatrin-treated animals (Wang et al. 2008) may explain the absence of major modifications in scotopic ERG amplitudes. However, this observation of plasticity in GES-treated mice indicates that taurine depletion induces a dysfunction at rod terminals.

Taurine is known to be a major neurotransmitter in the retina. It is present in bipolar cells and may have an effect on the third-order neurons: amacrine and ganglion cells. It has been shown showed that taurine suppresses the glutamatergic input in amacrine and ganglion cells through both ionotropic glutamate receptors and voltage-gated Ca^2+^ channel regulation (Bulley and Shen 2011). Since amacrine pathway is both photopic and scotopic (Anderson et al. 2011) and since amacrine cells seemed preserved in GES mice (Fig. 10), the lack of taurine itself may be responsible in part for the perturbation observed in the electrophysiologic response of GES mice, together with the photoreceptors loss.

At the level of retinal ganglion cells, we observed a significant loss induced by the GES treatment. This loss (19 %) was of the same amplitude as that of cone outer/inner segments (20 %) indicating thereby that both degenerative processes are undergoing in parallel and are not sequential as the retinal ganglion cell degeneration occurring in retinitis pigmentosa patients (Humayun et al. 1999) and animal models (Kolomiets et al. 2010). Even if ganglion cell loss was significant, ganglion cell degeneration was not sufficient to induce b-wave or oscillatory potential (OPs) alterations. Indeed, we could not find any difference in amplitude or latencies in both scotopic b-waves and OPs (Fig. 3a, d, e) between treated and untreated animals. This retinal ganglion cell degeneration in GES-treated mice was very similar to that obtained in vigabatrin-treated neonatal rats (Jammoul et al. 2010), which was already attributed to the taurine depletion. Therefore, our data on GES-treated animals indicate that taurine deficiency may indeed trigger both photoreceptor and retinal ganglion cell degeneration. This conclusion strengthens the hypothesis that taurine deficiency due to vigabatrin causes the retinal cell degeneration observed in vigabatrin-treated patients (Ravindran et al. 2001; Wild et al. 2006)

These results on the cellular dysfunction and degenerations induced by taurine deficiency could have relevance in human retinal physiopathology. Indeed, taurine depletion was recently reported in diabetic patients (Franconi et al. 1995) and taurine administration was proposed as a treatment for other complications of diabetes (Nakamura et al. 1999; Moloney et al. 2010). During diabetic retinopathy, retinal degeneration and in particular retinal ganglion cell loss may contribute to vision threatening (Barber 2003). The vascular dysfunction occurring during diabetic retinopathy is likely to reinforce the systemic reduction of taurine at the level of the retina. Furthermore, human cone photoreceptors were also reported to degenerate during diabetic retinopathy (Cho et al. 2000). The most relevant effect of taurine depletion could be at the level of retinal ganglion cells, which also undergo a degenerative process during diabetic retinopathy. A few recent studies have reported positive effect of taurine on retinal gliosis in diabetic animals (Yu et al. 2008; Zeng et al. 2009). Our study provides further support for the importance of taurine in retinal ganglion cell neuroprotection. Future studies will have to investigate further how taurine supplementation can interfere with retinal ganglion cell degeneration during diabetic retinopathy.

## Abbreviations

POS: Photoreceptor outer segments
ONL: Outer nuclear layer
INL: Inner nuclear layer
OPL: Outer plexiform layer
IPL: Inner plexiform layer
RGCL: Retinal ganglion cells layer
